# Molecular insights of a Unani formulation in targeting fibrosis-associated diseases

**DOI:** 10.3389/fphar.2026.1816025

**Published:** 2026-08-19

**Authors:** Rithika Thangaraju, Namrata Kamlesh, M. Shipin, Tridip Mitra, A. H. Sruthi Anil Kumar, Athira Gopinathan, Athar Parvez Ansari, Ritu Karwasra, Divyapriya Karthikeyan, Piyush Agrawal, Noman Anwar, P. Vineeth Daniel, Noor Zaheer Ahmed, Rajiv Janardhanan

**Affiliations:** 1 Division of Medical Research, Faculty of Medicine and Health Sciences, SRM Institute of Science and Technology, Kattankulathur, Tamil Nadu, India; 2 Department of General Surgery, Faculty of Medicine and Health Sciences, SRM Institute of Science and Technology, Kattankulathur, Tamil Nadu, India; 3 Department of Clinical Section, RRIUM, Chennai, Tamil Nadu, India; 4 Central Council for Research in Unani Medicine (CCRUM), Ministry of Ayush, Government of India, New Delhi, India

**Keywords:** anti-fibrotic property, cancer, drug formulation, ECM, fibrosis, phytochemicals, polyherbal unani formulation, therapeutic mechanism

## Abstract

Fibrosis is a pathological condition characterised by excessive deposition of the extracellular matrix (ECM) and persistent activation of myofibroblasts, and is associated with many chronic diseases. The association between fibrosis and cancer is increasingly recognised, where fibrosis contributes to the progression of cancer by increasing metastatic potential and resistance to therapy, thereby leading to poor clinical outcomes. A novel Polyherbal Unani Formulation (PUF), based on the Unani system of medicine, possesses a distinct phytochemical constitution with wide-spectrum therapeutic properties. Phytochemicals such as chebulinic acid, picrosides, flavonoids, and phenolic derivatives are reported to exert multi-targeted effects by attenuating oxidative stress, suppressing pro-fibrotic signalling pathways (TGF-β/Smad, NF-κB, PI3K/Akt), inhibiting epithelial-mesenchymal transition (EMT), and restoring fibroblast-to-myofibroblast plasticity. These individual compounds were systematically evaluated preclinically, establishing their effectiveness in modulating key molecular targets. Challenges such as phytochemical standardisation, bioavailability, and clinical validation remain significant for this distinct drug formulation, thereby improving its translational value. This review article considers PUF as a potential integrative therapeutic approach for fibrosis-related diseases and for aggressive cancers with fibrosis. In summary, we expect this PUF to target both tumour cells and the fibrotic tumour micro-environment, thereby integrating ethnopharmacological knowledge with molecular rationality.

## Graphical Abstract

**Figure:**
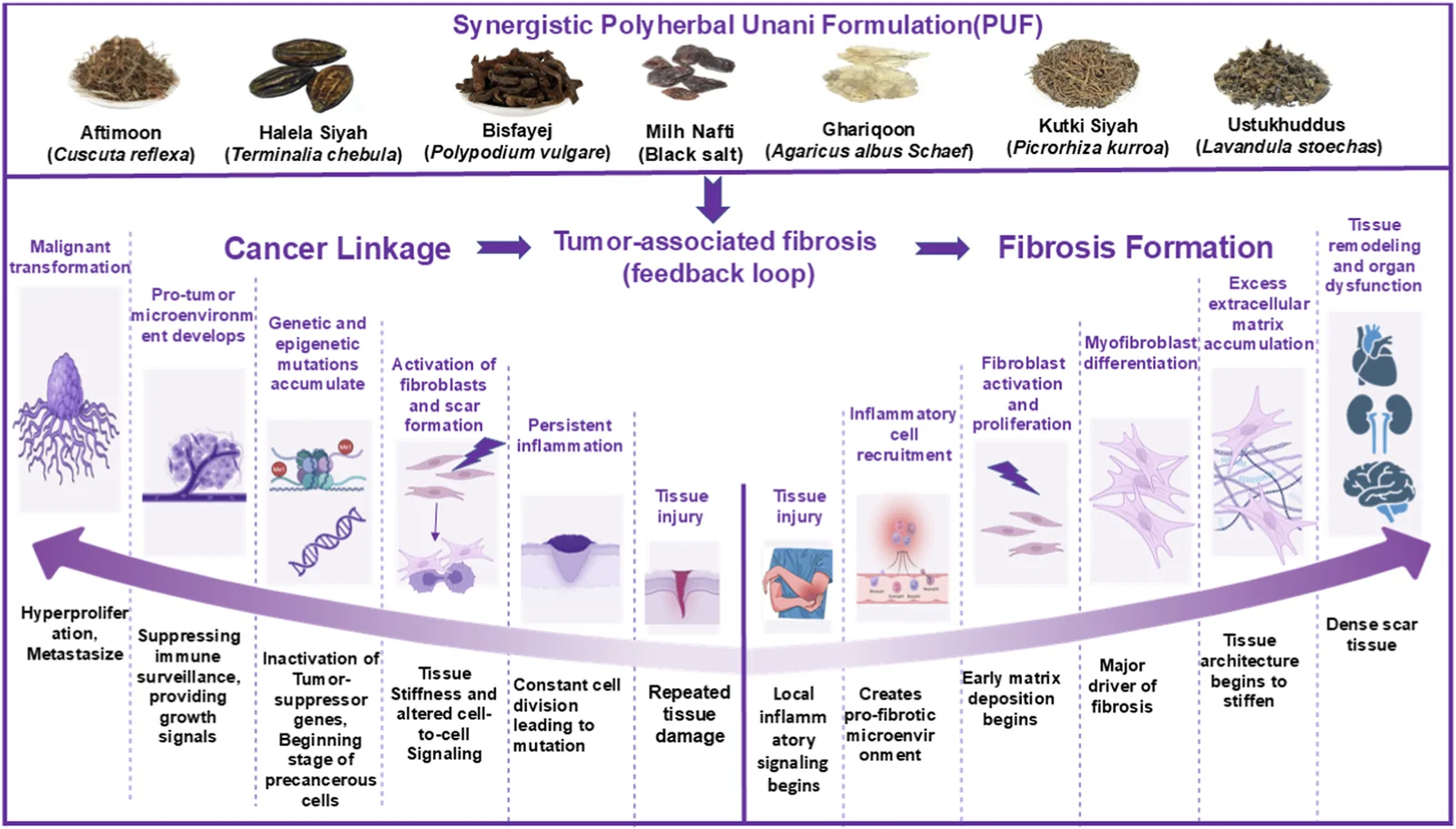
Schematic representation of Polyherbal Unani Formulation (PUF) disrupting the fibrosis feedback loop, from epigenetic surveillance and pro-cancer gene activation through inflammation and myofibroblast differentiation to ECM deposition and tissue remodelling.

## 1 Introduction

Fibrosis is a pathological process characterized by excessive deposition of extracellular matrix (ECM) components and persistent activation of myofibroblasts, leading to progressive tissue remodeling and organ dysfunction. It represents a common pathological endpoint in numerous chronic diseases affecting the liver, lungs, kidneys, heart, and other organs (Fuster-Martínez and Calatayud, 2024). Fibrosis-associated disorders contribute substantially to global morbidity and mortality, accounting for approximately 17%–18% of deaths worldwide and nearly 45% of deaths in developed countries (Sziksz et al., 2015). The growing burden of fibrosis is further reflected by increasing disability-adjusted life years (DALYs) reported across multiple organ systems over recent decades (Landolt et al., 2022). Despite differences in etiology, fibrotic diseases share common molecular and cellular mechanisms that drive progressive tissue scarring and functional decline.

Under physiological conditions, fibrosis originates as part of the normal wound-healing response, which involves the sequential phases of haemostasis, inflammation, proliferation, and tissue remodelling (Mutsaers et al., 1997). Following injury, platelets initiate clot formation while immune cells eliminate pathogens and cellular debris. During the proliferative phase, fibroblasts migrate to the site of injury and differentiate into myofibroblasts that synthesize ECM proteins required for tissue repair (Talbott et al., 2022). In healthy wound healing, these activated cells undergo apoptosis after repair is completed, allowing restoration of normal tissue architecture. However, when tissue injury persists or repair mechanisms become dysregulated, unresolved inflammation promotes continuous fibroblast activation and excessive ECM deposition, ultimately resulting in fibrosis (El Ayadi et al., 2020).

Chronic inflammation plays a central role in initiating and sustaining fibrogenesis. Unlike acute inflammation, which resolves following tissue repair, persistent inflammatory responses continuously stimulate immune cells to release pro-inflammatory cytokines and growth factors, including tumour necrosis factor-alpha (TNF-α), interleukin-1β (IL-1β), interleukin-6 (IL-6), and transforming growth factor-beta (TGF-β) (Artlett, 2013). Sustained exposure to these mediators promotes fibroblast activation and differentiation into ECM-producing myofibroblasts, resulting in excessive collagen deposition and progressive tissue stiffening (Younesi et al., 2024).

Fibrosis is regulated by a complex network of interconnected molecular pathways rather than a single signaling cascade. The TGF-β/Smad pathway acts as the principal driver of myofibroblast activation and collagen synthesis, whereas inflammatory pathways such as NF-κB amplify cytokine production and sustain chronic inflammatory responses. Excessive generation of reactive oxygen species (ROS) contributes to oxidative stress, which further potentiates TGF-β signalling and promotes cellular injury (Wynn, 2008). Simultaneously, EMT contributes to the expansion of the activated fibroblast population, while dysregulated ECM remodelling, characterized by excessive collagen deposition, altered matrix metalloproteinase (MMP) activity, and increased tissue stiffness, reinforces fibroblast activation (Radisky et al., 2007). Recent advances in single-cell sequencing, spatial transcriptomics, and multi-omics technologies have further revealed the involvement of diverse cellular populations, including fibroblasts, macrophages, endothelial cells, epithelial cells, and other stromal cells, highlighting fibrosis as a multifactorial disease driven by extensive cellular crosstalk and microenvironmental interactions (Feng et al., 2021; Chen et al., 2026; Kolostyak and Nagy, 2026; Xu et al., 2026).

Beyond its role in tissue scarring, fibrosis is increasingly recognized as a critical contributor to cancer initiation and progression. Persistent fibrosis creates a chronically inflamed and mechanically stiff microenvironment characterized by excessive ECM deposition, altered tissue architecture, and sustained cytokine signalling. These pathological changes promote genomic instability, cellular transformation, and tumour development (Rybinski et al., 2014; Wu et al., 2022). Within the tumour microenvironment, reduced oxygen availability creates a hypoxic state that stabilizes hypoxia-inducible factor-1 alpha (HIF-1α), a key transcriptional regulator of cellular adaptation to hypoxia (Hyun et al., 2024). Activated HIF signalling subsequently induces the expression of pro-angiogenic factors, particularly vascular endothelial growth factor (VEGF), resulting in pathological angiogenesis and vascular remodelling (Janardhanan et al., 2013; 2016; Nieves Torres et al., 2014; Yang et al., 2014). Persistent hypoxia also promotes fibroblast activation, EMT, immune evasion, metabolic reprogramming, and ECM remodelling, thereby reinforcing fibrotic progression and facilitating tumour invasion, metastasis, and therapeutic resistance (Zhao et al., 2021; Luo et al., 2022). Activated cancer-associated fibroblasts (CAFs) further contribute to this process by secreting cytokines, growth factors, and ECM components that sustain a reciprocal relationship between fibrosis and cancer progression (Rieder et al., 2025).

Despite significant advances in understanding the biology of fibrosis, currently available antifibrotic therapies primarily slow disease progression rather than reverse established fibrosis (Zhao X. et al., 2022). Approved agents such as pirfenidone and nintedanib provide clinical benefits in selected fibrotic disorders; however, their efficacy remains limited due to the complex and multifactorial nature of fibrogenesis. The involvement of multiple interconnected pathways, including inflammation, oxidative stress, ECM remodelling, EMT, and metabolic dysregulation, has stimulated growing interest in multitarget therapeutic approaches capable of simultaneously modulating several profibrotic mechanisms (Cox and Erler, 2014).

Traditional medicinal systems offer a unique opportunity to develop such multitarget therapies through the use of polyherbal formulations containing diverse bioactive constituents. According to the World Health Organization, a substantial proportion of the global population continues to rely on traditional medicine as a primary healthcare resource (Lee and Barnes, 2022). Among these approaches, the Polyherbal Unani Formulation (PUF) represents a promising candidate owing to its rich phytochemical composition and long history of therapeutic use. The constituent herbs of PUF contain flavonoids, phenolic compounds, tannins, terpenoids, and other bioactive molecules reported to possess antioxidant, anti-inflammatory, immunomodulatory, and antifibrotic properties (Husain et al., 2025). Collectively, these phytoconstituents are proposed to target key profibrotic pathways, including TGF-β/Smad, NF-κB, oxidative stress signalling, EMT, and ECM remodelling. This review therefore examines the mechanistic basis, current evidence, translational potential, and future prospects of PUF as a multi-target therapeutic strategy for fibrosis and fibrosis-associated diseases.

## 2 Polyherbal Unani formulation: phytochemical composition and multi-targeted therapeutic mechanisms

The Unani system of medicine is an ancient medical practice that traces its roots to ancient Greece, where the humoural philosophy was established by Hippocrates and further developed by the Arab and Persian scholars (Ali Khan et al., 2022). The Unani system of medicine was introduced into India during the eighth century and flourished extensively from the 12th century A.D. onwards under the patronage of the Delhi Sultanate and Mughal rulers, leading to its widespread acceptance and institutional development across the country (Government of India, 2016). At present, Unani medicine forms an integral component of the AYUSH system under the Ministry of Ayush, Government of India, supported through dedicated educational institutions, research councils, hospitals, and pharmaceutical industries that promote its clinical practice and scientific advancement (Ansari et al., 2024). It mainly focuses on the harmony of the four Humours (Blood, Phlegm, Yellow Bile, and Black Bile) and makes extensive use of herbal treatments that have been practiced for ages (El-Saadony et al., 2025). Classical Unani literature also emphasises the concept of Murakkab (compound) formulations, in which multiple medicinal ingredients are combined to achieve synergistic therapeutic effects. These formulations were traditionally designed to restore humoural balance, strengthen organ function, and enhance the body’s natural reparative capacity while minimising adverse effects (Mohd et al., 2019). However, in modern times, Unani medicine continues to hold significance owing to its holistic nature, relatively few side effects, and scientific investigations demonstrating the therapeutic efficacy of various Unani medicinal plants (Ali Khan et al., 2022). Recent studies have reported that commonly found medicinal plants such as Ashwagandha (*Withania somnifera*), Giloy (*Tinospora cordifolia*), and Black seed (*Nigella sativa*) possess anti-inflammatory, immunomodulating, and antioxidative effects, highlighting their possible applications in chronic ailments such as fibrotic and metabolic conditions (Karwasra et al., 2025). In recent years, increasing scientific interest in traditional systems of medicine has led to extensive phytochemical and pharmacological investigations of Unani medicinal plants. Several bioactive constituents, including flavonoids, alkaloids, phenolic compounds, terpenoids, tannins, and saponins, have been identified in these formulations and are reported to exhibit antioxidant, hepatoprotective, antifibrotic, and immunomodulatory activities through modulation of multiple cellular signalling pathways (El-Saadony et al., 2025).

Several clinical trials have been conducted to test the efficacy of various Unani polyherbal formulations, including “Tiryaq Wabai”, under randomised controlled conditions. A recently conducted placebo-controlled trial has demonstrated the immunomodulatory effects of these herbal formulations in patients with COVID-19 (Kumar et al., 2024). Moreover, several trials registered in the Clinical Trials Registry of India (CTRI) have investigated the use of Unani medicines as adjuvant or preventive therapy for various infectious conditions (Ahmad et al., 2026). Nevertheless, most of these studies are small and registered only at the regional level. Despite these limitations, research initiatives undertaken by institutions under the Ministry of Ayush and the Central Council for Research in Unani Medicine (CCRUM) have contributed substantially towards the drug standardisation, phytochemical characterisation, and experimental validation of several classical Unani formulations. Such efforts are strengthening the scientific basis of Unani medicine and facilitating its integration with contemporary biomedical research (Government of India, 2016).

In Unani medicine, this PUF synergistic effect represents the integrative heritage, comprising medicinal plants traditionally used to regulate tumours (Quamri, 2017), especially Khilt-e-Sawdā’ (Black Bile), which is implicated in cancer etiology and prognosis (Ahmed et al., 2016). The PUF is mentioned in the classical Unani textbook *Kamil-Us-Sana* within the chapter on *Saraṭān* (cancer) for its role in the evaluation and therapeutic management of *Khilt-e-Sawdā’* (Black Bile) (Husain et al., 2025). The PUF comprises seven principal medicinal herbal ingredients (Table 1), each containing multiple phytochemical constituents with distinct pharmacological activities. Collectively, these ingredients contribute to the proposed multi-target therapeutic potential of the complete formulation. These formulations are sourced from CCRUM, Ministry of Ayush, Government of India. These compositions may exert potent antifibrotic effects, restoring cellular homeostasis and supporting normal tissue architecture and function (Husain et al., 2025). Although mechanistic evidence for the complete PUF remains limited, available experimental observations suggest that its therapeutic activity is unlikely to arise from any single constituent. Rather, the formulation should be viewed as a systems-level intervention in which multiple phytoconstituents collectively modulate interconnected pathways involved in inflammation, oxidative stress, fibroblast activation, extracellular matrix remodelling, and immune regulation. Therefore, the following sections summarise the major constituents not as isolated therapeutic entities, but as contributors to the broader pharmacological network potentially underlying the activity of the complete formulation.

**TABLE 1:** Composition of the Polyherbal Unani Formulation (PUF). Table details individual composition names, botanical nomenclature, and plant part for each of the seven constituents. Milh Nafti (Kala namak) is a mineral salt devoid of botanical origin.

S. No	Ingredients	Botanical name	Part	Major phytochemical classes
1	Halela Siyah Hindi	*Terminalia chebula Retz.*	Fruit pulp	Hydrolysable tannins, chebulinic acid, chebulagic acid
2	Aftimoon Afriti	*Cuscuta reflexa Roxb.*	Stem	Flavonoids, phenolics
3	Bisfayej	*Polypodium vulgare L.*	Rhizome	Catechin, epicatechin, chlorogenic acid
4	Ustukhuddus	*Lavandula stoechas L.*	Flowers	Luteolin
5	Kutki Siyah	*Picrorhiza kurroa Royle ex Benth.*	Root	Picrosides
6	Ghariqoon	*Agaricus albus Schaef*	Fruiting body	Agaricic acid
7	Milh Nafti (Kala namak)	Not Applicable	-	Minerals

### 2.1 Halela siyah Hindi (terminalia chebula)

*Terminalia chebula* (*T. chebula*), the dried fruit of a member of the Combretaceae family, is one of the principal constituents of PUF. It is rich in hydrolysable tannins, including chebulic acid and chebulagic acid, which have been extensively investigated for their antioxidant, anti-inflammatory, and anti-fibrotic activities (Ahmad et al., 1998; Gupta, 2012; Khan et al., 2018). *Terminalia chebula* contains various phytochemicals, such as chebulic acid (CA), which counteract the fibrotic effects of glyceraldehyde-derived advanced glycation end products (AGEs) by dose-dependently reducing ROS production and collagen accumulation in LX-2 cells. Additionally, CA promotes ERK phosphorylation and enhances Nrf2 expression, mechanisms relevant to mitigating fibrosis in conditions such as non-alcoholic steatohepatitis (NASH) (Naik et al., 2004; Suchalatha et al., 2005; Suresh et al., 2011). Another compound, chebulagic acid from *T. chebula,* shows strong anti-proliferative effects on retinoblastoma cells by inducing G1 arrest, inhibiting NF-κB, and triggering apoptosis (Kumar et al., 2014). *Terminalia chebula* extract demonstrates nephroprotective effects in hyperuricemic nephropathy (HN) by exerting anti-inflammatory actions and ameliorating fibrosis through modulation of the TLR4/MyD88/NF-κB signalling pathway, supporting its broader therapeutic potential in managing HN and other chronic kidney diseases (Liu et al., 2024). Phytochemicals from *T. chebula* are known to inhibit histamine secretion (El-Shamarka et al., 2024) as histamine, through H_1_ receptors, promotes fibroblast proliferation, TGF-β secretion, and collagen synthesis. By blocking histamine release, these acids may help reduce fibrotic responses (Veerappan et al., 2013). Preclinical studies indicate that *T. chebula* has a favourable safety profile, with no significant toxicity observed under the tested experimental conditions. However, comprehensive safety evaluation of the complete PUF remains necessary (Suganthy et al., 2018; Bulbul et al., 2022).

### 2.2 Aftimoon afriti (*Cuscuta reflexa*)

Aftimoon (*Cuscuta reflexa*) contains phenolic compounds, flavonoids, and other bioactive constituents that exhibit antioxidant, anti-inflammatory, and anti-fibrotic activities. These properties are particularly relevant to fibrosis because they suppress oxidative stress, inflammatory signalling, and extracellular matrix accumulation (Bulbul et al., 2022). Studies have shown that phytochemicals derived from *C. reflexa* can suppress fibroblast activation and ECM accumulation. Ethyl acetate and acetone extracts of *C. reflexa* exhibit significant antitumour activity against lung cancer (H-1299) and breast cancer (MCF-7) cell lines. These extracts were found to contain various antioxidant molecules (Ali et al., 2020). The presence of phenols, polyphenols, and flavonoids in *C. reflexa* has demonstrated anti-cytotoxic, anti-inflammatory, hepatoprotective, and antioxidant activities, which are relevant to the pathological features of fibrotic conditions (Udavant et al., 2012; Pullaiah and Ramaiah, 2021). Furthermore, the methanolic and ethyl acetate fraction of *C. reflexa* shows significant inhibitory activity against the α-glucosidase enzyme, thereby delaying glucose absorption into the bloodstream (Anis et al., 2002), eventually reducing fibroblast activation by inactivation of profibrotic signalling pathways (TGF-β, CTGF, and other fibrotic cytokines) and slowing ECM accumulation, indirectly curbing fibrosis progression.

### 2.3 Bisfayej (*Polypodium vulgare L.*)

*Polypodium vulgare L.* contains flavonoids and phenolic acids, particularly epicatechin, catechin, and chlorogenic acid, that contribute to its antioxidant and anti-fibrotic activities (Farràs et al., 2021). *Polypodium vulgare* contains several bioactive compounds that are believed to contribute to its broad therapeutic potential. Epicatechin and catechin (flavonoids) are well-known for anti-fibrotic, anti-inflammatory, and antioxidant effects (Shariati et al., 2019; Kim and Heo, 2022). Chlorogenic acid (CGA, 5-O-caffeoylquinic acid) can inhibit pathways such as NF-κB, TGF-β, and MMPs, which are implicated in fibrosis progression (Yang et al., 2017). In summary, by modulating fibroblast function, reducing oxidative stress, and inhibiting excessive extracellular matrix deposition, these compounds help prevent or attenuate fibrotic progression (Albrakati, 2025).

### 2.4 Ustukhuddus (*Lavandula stoechas L.)*

*Lavandula stoechas L.* is an aromatic medicinal herb whose principal bioactive flavonoid, luteolin, has attracted considerable attention because of its anti-inflammatory and anti-fibrotic properties (Algieri et al., 2016). A study by Li *et al.*, demonstrated that luteolin attenuates liver fibrosis by suppressing the activation, proliferation, migration, collagen synthesis, and fibrosis-related gene expression of activated hepatic stellate cells under TGF-β1 or PDGF stimulation. Mechanistically, luteolin inhibited TGF-β/Smad and PI3K/Akt signalling, reducing hepatic stellate cell activation and extracellular matrix production, while promoting apoptosis and G1-phase arrest. *In vivo*, luteolin alleviated hepatic fibrosis and reduced phosphorylated Smad2 and Akt expression. Accordingly, the description of luteolin has been revised to accurately reflect its established anti-fibrotic mechanism rather than implying mechanisms primarily associated with cancer biology (Li et al., 2015).

### 2.5 Kutki siyah *(Picrorhiza kurroa)*

*Picrorhiza kurroa Royle*., is a well-known medicinal and modern herb (Soni and Grover, 2019). Kutkoside, pikuroside, kutkin, bartsioside, boschnaloside, picroside V, and mussaenoidic are seven distinct glucosides, collectively called “iridoid glycosides,” which give *Picrorhiza kurroa* its unique medicinal properties. Picroside I treatment for hepatic fibrosis helps regulate energy and lipid metabolism, inhibits abnormal blood vessel formation (pathological angiogenesis) through the sphingolipid pathway, and modulates bile acid production by reducing bile acid levels and increasing the activity of the enzyme CYP8B1 via the PPAR signalling pathway (Song et al., 2016; Xiong et al., 2020). Another target is Annexin A2 (Anxa2), a calcium-dependent phospholipid-binding protein that promotes plasmin production. Plasmin, in turn, can release fibroblast growth factors or activate TGF-β1, thereby amplifying inflammation and driving the fibrosis process (Brownstein et al., 2004). Within the complete PUF, these activities are expected to complement those of the remaining constituents by targeting oxidative stress, inflammatory signalling, and extracellular matrix remodelling. However, direct experimental validation of these synergistic interactions remains limited.

### 2.6 Ghariqoon (*Agaricus albus schaef*.)

*Agaricus albus Schaef.*, also known as the female agaric or pharmacist’s agaric, is a large, fragile mushroom. The presence of agaricic acid or laricic acid (α-cetylcitric acid) contributes to the therapeutic effect (Hanson, 2008) by targeting mitochondrial dynamics. It interacts with adenine nucleotide translocase (ANT) and induces opening of the mitochondrial permeability transition pore (MPTP), which initiates mitochondrial membrane potential collapse, calcium efflux, and apoptosis (Chen et al., 2023). This mechanism facilitates the removal of apoptosis-resistant myofibroblasts, disrupts profibrotic calcium signalling in fibrosis, and promotes cancer cell death by disrupting energy metabolism and enhancing oxidative stress, positioning it as a promising mitochondrial-targeting compound for both anti-fibrotic and anti-cancer applications.

### 2.7 *Milh Nafti* (black salt)

Within PUF, Kala Namak (Milh Nafti) is believed to serve primarily as a supportive component rather than a direct anti-fibrotic agent. Its potential role lies in improving gastrointestinal function and enhancing the absorption and bioavailability of co-administered phytoconstituents, while hydrogen sulfide released from the salt may contribute to the modulation of oxidative stress and fibroblast activation (Bali and Khan, 2024). Kala Namak enhances therapeutic efficacy by improving gastrointestinal function, increasing the absorption and bioavailability of phytoconstituents, and promoting the elimination of metabolic waste. The PUF helps restore humoural balance (Tadheel-e-Mizaj) and prevent the accumulation of morbid matter (Madda Fasida), which are considered the underlying causes of chronic diseases (Husain et al., 2025). Although not directly cytotoxic or anti-fibrotic, Kala Namak may contribute to the formulation by modulating gastrointestinal physiology and electrolyte balance, thereby influencing the absorption and bioavailability of co-administered phytoconstituents. Through this supportive role, it may indirectly enhance the overall efficacy of key therapeutic components such as *C. reflexa, T*. *chebula,* and *P*. *kurroa* (Bali and Khan, 2024). By reducing oxidative stress, improving nitric oxide levels, and modulating the gut microbiome, Kala Namak’s multifaceted role highlights the importance of integrating traditional knowledge with modern research to explore its potential in anti-fibrotic and anti-cancer strategies.

### 2.8 Integrative action of PUF components

The therapeutic efficacy of this PUF arises from a sophisticated integrative synergy in which individual components converge at multiple nodes of the fibrotic signalling network. While *T. chebula* (chebulinic acid) and *L. stoechas* (luteolin) directly target the “driver” of tissue stiffening by inhibiting TGF-β/Smad2/3 phosphorylation and inducing apoptosis in activated myofibroblasts (Li et al., 2015; Feng et al., 2021), they are comparatively complemented by *P. kurroa* (picrosides) and *C. reflexa*. These latter components act further upstream by suppressing the NF-κB and TNF-α-mediated inflammatory triggers that initiate the transition from acute injury to chronic fibrosis (Suresh et al., 2011; Xiong et al., 2020). Integration is also evident in the redox-defence axis; *T. chebula* enhances endogenous Nrf2-mediated antioxidant enzymes, while *C. reflexa* provides immediate radical scavenging, creating a dual-layered protection against the oxidative stress that fuels ECM deposition. Furthermore, the inclusion of Black Salt serves a vital integrative role, not through direct anti-fibrotic activity but as a bio-enhancer that improves the absorption and bioavailability of the primary phytoconstituents (Bali and Khan, 2024). Collectively, these complementary mechanisms suggest that PUF may interrupt the self-perpetuating fibrosis feedback loop at multiple stages, including inflammatory activation, oxidative stress, metabolic dysregulation, and extracellular matrix remodelling. This integrated mode of action provides a mechanistic rationale for the potential therapeutic advantage of the complete formulation over individual phytochemicals, although direct experimental validation of these synergistic interactions remains limited (Williamson, 2001; Wagner, 2011; Caesar and Cech, 2019) (Table 2).

**TABLE 2:** Functional comparison of PUF components in the fibrosis feedback loop.

Phase of fibrosis	Primary component(s)	Specific molecular target	Comparative advantage	References
Initial Injury	*P. kurroa*, *C. reflexa*	NF-κB, TNF-α, IL-6	Ameliorates the transition from acute injury to chronic inflammation.	Suresh et al. (2011), Garg et al. (2025), Ren et al. (2026)
Myofibroblast Activation	*T. chebula*, *L. stoechas*	TGF-β/Smad, PDGF	Directly blocks the “driver” of tissue stiffening.	Weiskirchen (2016), gianni (2018)
ECM Remodeling	*P. vulgare*, *P. kurroa*	MMP-2, MMP-9, TIMPs	Restores the balance between ECM production and degradation.	Garg et al. (2025)
Metabolic Shift	*P. kurroa*, *A. albus*	AMPK, Mitochondria	Induces AMPK-mediated reprogramming, reducing glycolytic flux and restoring mitochondrial oxidative metabolism, thereby limiting myofibroblast activation and survival	Kui et al. (2025)

## 3 Intersection of PUF as a plausible therapy for anti-fibrotic mechanisms

**FIGURE 1:**
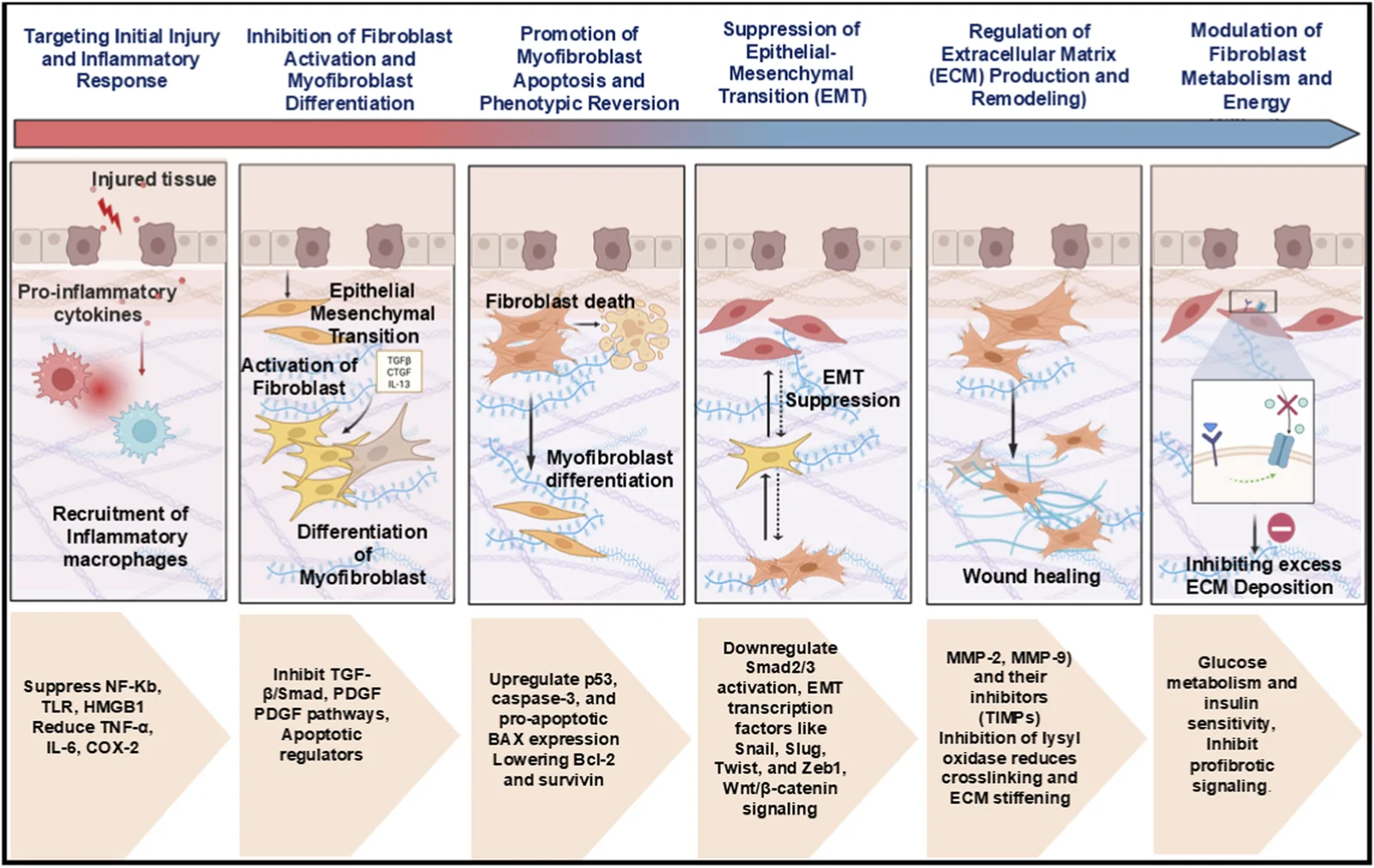
Schematic representation of key therapeutic targets for antifibrotic interventions across multiple stages of fibrosis pathogenesis. The diagram illustrates five interconnected phases: (1) targeting initial injury response (inhibiting NF-κB, IL-6, COX-2 in cytokine-producing macrophages); (2) Inhibition of myofibroblast differentiation (Smad/PDGF pathway regulators); (3) promotion of myofibroblast phenotypic reversal and apoptosis (caspase-3/8, Bax expression, survivin downregulation); (4) suppression of epithelial-mesenchymal transition (EMT; Smad2/4, Twist-1/2, Slug transcription factors); and (5) Regulation of extracellular matrix (ECM) remodelling and fibroblast energy utilization (MP-2/9 inhibitors, lysyl oxidase, TIMPs, glucose metabolism inhibitors). Wound-healing modulation and excess ECM deposition are highlighted as critical resolution points.

PUF comprises seven key compounds that collectively engage a broad array of anti-fibrotic therapeutic targets aligned with advanced contemporary therapeutic strategies for fibrosis and related cancers (Figure 1). The mechanistic effects discussed in this section are derived primarily from preclinical studies on individual phytochemical constituents or herbal ingredients of PUF. Direct experimental evidence evaluating the complete PUF formulation remains limited (Wagner, 2011; Caesar and Cech, 2019).

### 3.1 Targeting initial injury and inflammatory response

The initial inflammatory response following tissue injury is a critical determinant of fibrosis, as persistent innate immune activation drives the transition from normal wound healing to chronic fibrogenesis. Based on available studies of individual phytoconstituents, PUF may collectively suppress early innate immune activation by targeting complementary inflammatory pathways. However, this integrated effect has not yet been experimentally validated using the complete formulation. For instance, picrosides inhibit NF-κB signalling and pro-inflammatory cytokines such as TNF-α and IL-6, reducing inflammatory cell infiltration and ROS generation by activating antioxidant defence pathways (Ma and Shi, 2021; Yao et al., 2022; Soni et al., 2024). This effectively reduces acute injury severity and prevents the transition to chronic inflammation, a key step that propels fibroblast activation (El Ayadi et al., 2020). *Cuscuta reflexa* similarly modulates inflammatory cytokines and cyclooxygenase-2 (COX-2), an enzyme that plays a key role in inflammation and pain, to anti-inflammatory effects at the early injury phase (Suresh et al., 2011; Adnan et al., 2020). Such redox and cytokine modulation aligns with modern therapeutic strategies aiming to blunt inflammatory-triggered fibrogenesis. Targeting post-injury sterile inflammation pathways through high-mobility group box 1 (HMGB1) and Toll-like receptor (TLR is gaining attention; picrosides are reported to inhibit HMGB1-RAGE and TLR-4-NF-κB signaling by natural modulation which prevents inflammatory cytokine production and fibrotic tissue remodeling (Li et al., 2016) (Table 3).

**TABLE 3:** Pharmacological activities of key Unani medicinal plants and their active phytochemicals.

Plant	Plant part	Active ingredients (phytochemicals)	Action	Key molecular targets	Study type	Reference
Halela Siyah Hindi *(T. chebula Retz.)*	Fruit extract	Chebulinic acid (hydrolysable tannin)	Hepatoprotective via antioxidant and Nrf2 pathway activation	↓ ROS, ↓ LDH; ↑ HO-1, ↑ NQO1; *in vivo* ↓ ALT, AST, MDA; ↑ SOD; histopathology improved	*In vitro* (L-02 hepatocytes) + *In vivo* (zebrafish, mice)	Feng et al. (2021)
	Fruit extract	Aqueous tannin fraction	Antioxidant, hepatoprotective	↓ ALT, AST, LDH; ↓ GSSG, lipid peroxidation; reduced histological damage	*In vitro* (rat hepatocytes) + *In vivo* (rats)	Lee et al. (2005)
	Fruit pericarp	Hydrolysable tannin fraction (HTF) including chebulagic acid, corilagin	Anti-inflammatory via NF-κB/MAPK inhibition	↓ NO, ↓ ROS, ↓ TNF-α, COX-2, iNOS; ↓ p65, p38, pERK protein and mRNA	*In vitro* (RAW 264.7 cells)	Ekambaram et al. (2022)
	Fruit pericarp	HTF (tannins)	Reduces collagen-mediated inflammation	↓ Arthritic score, paw edema, spleen index; ↓ serum/joint cytokines	*In vivo* (collagen-induced arthritis in mice)	Ekambaram et al. (2020)
	Fruit	Methanol extract of chebulinic acid, tannic acid and ellagic acid	Anti-cancerous (Growth inhibitory)	G1 cell cycle arrest, ↓ oncogenic pathways NF-κB and Cyclin D1, ↑ apoptotic markers like caspase-3 and p53	*In vitro* Human (MCF-7) and mouse (S115) breast cancer cell line, human osteosarcoma cell line (HOS-1), human prostate cancer cell line (PC-3) and non-tumorigenic, immortalized human prostate cell line (PNT1A)	Saleem et al. (2002)
	Fruit (standardized water extract)	Polyphenols, tannins, triterpenes and flavonoids (such as 2,4-chebulyl- β-D-glucopyranose, 1,6-di-*O*-galloyl-β-D-glucose, quercetin, gallic acid, caffeoylquinic acid, chebulinic acid, casuarinin, and chelanin)	Antioxidant and Antitumourigenic: Free radical scavenging, tumor growth inhibition	↑ DPPH and ABTS scavenging; ↑ SOD, CAT, GSH; ↓ Tumor cell viability, ↑ Apoptosis	*In vivo* (Mice) and *In vitro* (U-937)	Na Takuathung et al. (2023)
Aftimoon Afriti (*C. reflexa Roxb.*)	Stem	Aqueous extract	anti-inflammatory and anti-cancer activities	↑ p53, BAX ↓Bcl-2, surviving, and interplay in TNF-α, COX-2 and NF-κB signalling	*In vitro* (murine macrophage cell line RAW264.7 and Hep3B cells)	Suresh et al. (2011), Ansari et al. (2020)
	Whole Plant	Chloroform and ethanol extract	Antitumour activity	↓ Tumor volume and viable cell count	*In vivo* (Swiss albino mice)	Chatterjee et al. (2011)
Bisfayej (*P. vulgare L.*)	Fronds	Methanolic extract	Potential chemopreventive	Non-cytotoxic	*In vitro* (NIH 3T3, HaCaT, HeLa, HepG2, MCF-7 and A549.	Farràs et al. (2021)
	Rhizome	Flavonoids	Anticancer	↑ Apoptosis	*In vitro* (A375 melanoma cells)	Tabeshpour et al. (2023)
Ustukhuddus (*L. stoechas L.)*	Whole plant	Methanol extract, Phytosterols	Anticancer effect	↓ Cell survival	*In vivo* (HepG2 cell line)	Siddiqui et al. (2020)
Kutki Siyah *(Picrorhiza kurroa)*	Rhizome	Picroside II (iridoid glycoside)	Anti-migration, anti-invasion, anti-angiogenesis (anti-tumor)	Downregulation of VEGF, MMP-2, MMP-9; suppression of tumor cell motility pathways	*In vitro* (tumor cell lines); *In vivo* (xenograft mouse model)	Lou et al. (2019)
	Rhizome	Iridoid glucosides such as picroside I, picroside II, picroside III, picroside IV, kutkoside, pikuroside and flavonoids like apocynin and vanillic acid	Antioxidant, protection against DNA/protein damage	Scavenging free radicals; reducing lipid peroxidation; protecting DNA from oxidative stress	*In vitro* antioxidant assays (DPPH, ABTS, FRAP); DNA/protein protection assays	Krupashree et al. (2014)
	Rhizome	New iridoid glycosides (picrosides), phenylethanoid glycosides, cucurbitacins	Hepatoprotective protects hepatocytes from toxin-induced injury	Reduced D-Gain-induced cytotoxicity without blocking LPS-driven macrophage activation and lowered hepatocyte sensitivity to TNF-α	*In vitro* hepatocyte model, TNF-α)-induced cytotoxicity in L929 cells; *In vivo* (rat hepatotoxicity model, CCl_4_)	Sakamoto et al. (2023)

This table summarizes the plant species, specific plant parts, major active ingredients (phytochemicals), primary pharmacological actions, key molecular targets or mechanisms (functional upregulation and downregulations are indicated by arrows), study types (*in vitro, in vivo*, or both), and corresponding references. Data highlight antioxidant, anti-inflammatory, hepatoprotective, and anticancer effects, particularly relevant for fibrosis-related research, drawn from preclinical studies across various models including cell lines (RAW 264.7, HepG2, MCF-7), animal models (mice, rats, zebrafish), and biochemical assays.

Persistent inflammation not only exacerbates tissue damage but also activates profibrotic signaling pathways that drive fibroblast activation and excessive ECM deposition, ultimately resulting in fibrosis. Among the various signaling pathways implicated in fibrogenesis, TGF-β is recognized as the master regulator and serves as a critical link between chronic inflammation, fibrosis, and tumorigenesis (Zhao X. et al., 2022). Sustained activation or dysregulation of TGF-β signaling promotes fibroblast activation, myofibroblast differentiation, and ECM accumulation, while also facilitating EMT, immune evasion, angiogenesis, and metastasis during cancer progression. Consequently, targeting the TGF-β signaling pathway has emerged as a promising therapeutic strategy for the treatment of fibrosis and fibrosis-associated cancers (Xiao and Puré, 2025).

### 3.2 Inhibition of fibroblast activation and myofibroblast differentiation

Central profibrotic signaling cascades are inhibited by phytochemicals of PUF, notably TGF-β/Smad and PDGF pathways key drivers of fibroblast-to-myofibroblast differentiation and proliferation, hallmarks of fibrosis progression (Jiao et al., 2025). Evidence from studies of individual phytoconstituents suggests that the complete PUF may collectively target these pathways through complementary mechanisms. The chebulinic acid modulates apoptotic regulators while reducing fibroblast proliferation markers (Chuang et al., 2011). Furthermore, inhibition of NF-κB signaling reduces the perpetuation of inflammatory and proliferative cues within fibrotic tissue. Recent evidence suggests that naturally derived bioactive compounds can regulate TGF-β/Smad signalling through multiple complementary mechanisms rather than acting on a single molecular target (Song et al., 2026). Besides suppressing fibroblast activation, many phytochemicals simultaneously reduce oxidative stress, dampen inflammatory responses, and influence epigenetic regulators involved in fibrogenesis. This broad-spectrum mode of action more closely reflects the complex biology of fibrosis and provides additional support for investigating multi-component herbal formulations such as PUF as potential anti-fibrotic therapies (Chen et al., 2025). These multi-axis inhibitory effects mirror the mechanisms targeted by emerging synthetic antifibrotics currently in clinical trials for IPF and liver fibrosis (Bonella et al., 2023).

Considerable progress has recently been made in developing TGF-β inhibitors, including monoclonal antibodies, ligand traps, receptor kinase inhibitors, antisense oligonucleotides, and combination regimens with immune checkpoint blockade. Although several agents remain under clinical investigation, these approaches illustrate the growing clinical interest in selectively modulating TGF-β signaling while minimizing systemic toxicity (Jing et al., 2025).

### 3.3 Promotion of myofibroblast apoptosis and phenotypic reversion

Persistent myofibroblasts resist apoptosis and sustains fibrosis (Hinz and Lagares, 2020). Chebulinic acid and related compounds promote apoptosis by increasing p53, caspase-3, and pro-apoptotic BAX expression while lowering Bcl-2 and survivin, disrupting survival pathways (McElhinney et al., 2023). These effects coincide with enhanced antioxidant status, reducing ROS that otherwise maintain fibrotic cell survival (Lagares et al., 2017). Picroside II additionally modulates macrophage phenotypes towards an anti-fibrotic profile, further supporting the resolution of fibrosis. This apoptosis induction is in line with the therapeutic approach that uses BH3 mimetics and agents that promote apoptosis. Epigenetic modifiers (HDAC inhibitors) and PPAR-γ agonists complement pro-apoptotic approaches by reprogramming myofibroblasts into quiescence, a concept that is also explored in PUF ingredients (Abbasi et al., 2024).

### 3.4 Suppression of epithelial-mesenchymal transition (EMT)

PUF-type compounds may block the phosphorylation of TGF-β receptors and the activation of Smad2/3 (Lan et al., 2025). They also suppress the transcription factors of EMT, such as Snail, Slug, Twist, and Zeb1, which prevent the reduction of epithelial properties (E-cadherin) and the induction of mesenchymal properties (N-cadherin, vimentin). Quercetin and ellagic acid have shown the suppression of EMT in lung and kidney fibrosis models, consistent with their position in the PUF (Saitoh, 2023; Lan et al., 2025). The inhibition of EMT is still an emerging target, with drugs targeting the interruption of TGF-β and Wnt/β-catenin signaling pathways, which can potentially prevent this harmful cellular transition.

### 3.5 Regulation of extracellular matrix (ECM) production and remodeling

ECM deposition plays a vital role in fibrosis formation where PUF synergistic compound effect reduce fibrotic ECM protein expression (collagens I and III, fibronectin) through TGF-β pathway inhibition and suppress pro-inflammatory cytokines (IL-6, TNF-α), which exacerbate ECM synthesis (Wight and Potter-Perigo, 2011). They also regulate matrix metalloproteinases (MMP-2, MMP-9) and their inhibitors (TIMPs), maintaining ECM turnover balance (Zhao X. et al., 2022). Importantly, inhibition of lysyl oxidase reduces crosslinking and ECM stiffening, processes that foster fibrosis and tumor progression (Zhao X. et al., 2022). Therapeutic targeting of post-translational modifications of ECM proteins and MMP activity is the most promising approach, with new inhibitors in clinical trials, mirroring the effects of natural compounds.

### 3.6 Modulation of fibroblast metabolism and energy utilization

Activated myofibroblasts undergo metabolic reprogramming characterized by increased glycolysis, glutaminolysis, and altered mitochondrial function to sustain their biosynthetic and contractile activities. Targeting these metabolic alterations represents a promising anti-fibrotic strategy. Phytoconstituents present in PUF may modulate key metabolic regulators such as AMP-activated protein kinase (AMPK), thereby reducing glycolytic dependence and promoting mitochondrial oxidative phosphorylation (Li et al., 2022; Zhao X. et al., 2022). This shift in cellular metabolism can limit myofibroblast activation, proliferation, and ECM production (Kumari et al., 2021). Additionally, restoration of mitochondrial homeostasis and reduction of reactive oxygen species (ROS) further contribute to the attenuation of fibrogenic signaling pathways. Collectively, these effects suggest that metabolic modulation is a critical component of the anti-fibrotic activity of PUF (Kreutzer et al., 2022).

### 3.7 Immune microenvironment modulation and checkpoint inhibition

The immunosuppressive tumour microenvironment plays a critical role in tumour progression and expansion, which could be hindered by PUF compounds, which display strong binding affinity for the PD-1/PD-L1 immune checkpoint, indicating their potential to restore T-cell functionality and enhance immune surveillance that is repressed during fibrosis and tumorigenesis (Carter et al., 2023). CAFs are now recognised as active regulators of tumour progression rather than merely structural components of the tumour stroma. Beyond producing extracellular matrix proteins, CAFs communicate extensively with immune cells, endothelial cells, and cancer cells to promote angiogenesis, metabolic adaptation, immune suppression, and therapeutic resistance. Consequently, strategies that interfere with CAF-mediated signalling are increasingly regarded as promising approaches for simultaneously limiting fibrosis and tumour progression (Guo and Xu, 2024). By reducing the infiltration of regulatory T cells (Tregs) and M2 macrophages, they can disrupt the fibrotic and immunosuppressive microenvironment (Davidsson et al., 2020). The combination of antifibrotic mechanisms and immune checkpoint blockade is a cutting-edge approach, and the PUF compounds are a promising natural multi-target adjunct.

## 4 Other fibrotic disease targets of PUF

In addition to its role in cancer therapy, based primarily on studies of its individual 451 phytochemical constituents, PUF may possess therapeutic potential for multiple fibrotic 452 disorders. For instance, bioactive constituents such as picroside I and Chebulic acid have demonstrated antifibrotic potential in terms of hepatic fibrosis by modulating key metabolic and signalling pathways implicated in fibrogenesis. A study by Xiong et al. reported that picroside I reduced liver fibrosis in mice by regulating sphingolipid metabolism, bile acid biosynthesis, and PPAR signalling pathways (Xiong et al., 2020). Chebulic acid, another phytochemical isolated from *T. chebula Retz*., reduces hepatic fibrosis by inhibiting advanced glycation end-product-induced oxidative stress and collagen accumulation in hepatic stellate cells through activation of the ERK-mediated Nrf2 signalling pathway, thereby enhancing antioxidant defences and suppressing fibrogenic responses (Koo et al., 2016). Ma et al. (2025) highlighted the significant antifibrotic potential of natural products derived from marine and terrestrial sources. It discusses various classes of compounds, such as flavonoids, saponins, polyphenols, terpenoids, and natural polysaccharides, that act through key signalling pathways, including TGF-β/Smad, Nrf2, NF-κB, and AMPK. These compounds show both preventive and therapeutic effects in pulmonary fibrosis and IPF (Ma et al., 2025). In a study by Liu et al., *T*. *chebula Retz.* The extract was found to reduce kidney injury markers and improve renal pathology in experimental models, while significantly downregulating inflammatory mediators and fibrotic genes. It inhibits the NF-κB signaling pathway via the TLR4/MyDli pathway, decreasing cytokines and extracellular matrix proteins. These effects suggest potential antifibrotic activity by suppressing inflammation and preventing fibrosis-related tissue remodeling (Liu et al., 2024). *Lavandula stoechas* flower extract demonstrated cardioprotective effects in isoprenaline-induced myocardial injury by significantly reducing cardiac enzymes, triglycerides, and tissue damage (Siddiqui et al., 2023). These effects, along with histological improvement, suggest potential relevance to cardiac fibrosis, as the extract may limit inflammation and tissue remodeling processes that contribute to fibrotic progression (Kong et al., 2014; Henderson et al., 2020). The ability of PUF to regulate cell death, maintain immune balance, and influence epigenetic mechanisms highlights its wide-ranging therapeutic potential across fibrotic conditions affecting different organs. Phytochemicals derived from plants have additional supportive evidence from other types of fibrotic conditions as well (Song et al., 2026). In the case of an experimental model for the development of fibrosis due to inflammatory bowel disease, different types of phytochemicals such as flavonoids, polyphenols, terpenoids, and alkaloids have exhibited anti-fibrotic properties through the regulation of TGF-β/Smad, NF-κB, Wnt/β-catenin, and oxidative stress signaling pathways in order to prevent the activation of myofibroblasts and excessive accumulation of extracellular matrix. This evidence proves the wide range of applications of phytochemical therapy in various fibrotic conditions (Younesi et al., 2024). Overall, these multifaceted actions suggest that PUF could serve as a promising anti-fibrotic agent, though preclinical and clinical studies are still needed to confirm its efficacy.

## 5 Experimental models for fibrosis: *in vitro* and *in vivo* key molecular targets

Most of the available evidence summarized here originates from *in vitro* experiments and animal studies evaluating individual phytochemical constituents. Clinical evidence for the complete PUF formulation in fibrosis-related diseases is currently lacking, highlighting the need for well-designed translational and clinical investigations. *In vitro* studies using cell lines such as RAW 264.7 macrophages, MCF-7, PC-3, and HepG2 have shown reductions in oxidative stress markers (ROS, LDH), inflammatory mediators (TNF-α, COX-2, iNOS), and pro-oncogenic proteins (NF-κB, Cyclin D1), along with increased expression of antioxidant enzymes (HO-1,NQO1, SOD, GSH) and pro-apoptotic markers p53, caspase-3, BAX) (Saleem et al., 2002; Farràs et al., 2021; Ekambaram et al., 2022). *In vivo* experiments on rodents and zebrafish models confirm the validity of substantial expression in liver function markers (decreased levels of ALT, AST, MDA), reduction in histological damage, and tumor inhibition, as well as enhanced apoptosis and lowest toxicity (Lee et al., 2005; Feng et al., 2021). Results of these *in vitro* and *in vivo* experiments offer a rationale for future clinical studies. The unavailability of these results creates a demand for well-designed large-scale clinical trials to demonstrate its therapeutic efficacy and safe incorporation into fibrosis management protocols.

Together, these evidences point out that the mechanism behind the therapeutic effect of PUF is due to the synergistic action of regulating several different pathways instead of targeting only one. The phytoconstituents in the formulation affect oxidative stress, chronic inflammation, TGF-β/Smad and NF-κB pathways, EMT, ECM remodeling, and immunity. These are some of the processes associated with the development of fibrosis (White et al., 2022). It is because of these diverse effects that the possibility of using the PUF formulation as a treatment approach for fibrosis and malignancies related to fibrosis should be considered from a mechanistic perspective. It is important to note that most evidence available comes from experiments involving isolated phytochemicals. Thus, there is a need for pharmacological studies and clinical trials on the complete formulation to ascertain its efficacy (White et al., 2022; Fuster-Martínez and Calatayud, 2024; Chen et al., 2025).

## 6 Challenges and future directions in drug formulations

**FIGURE 2:**
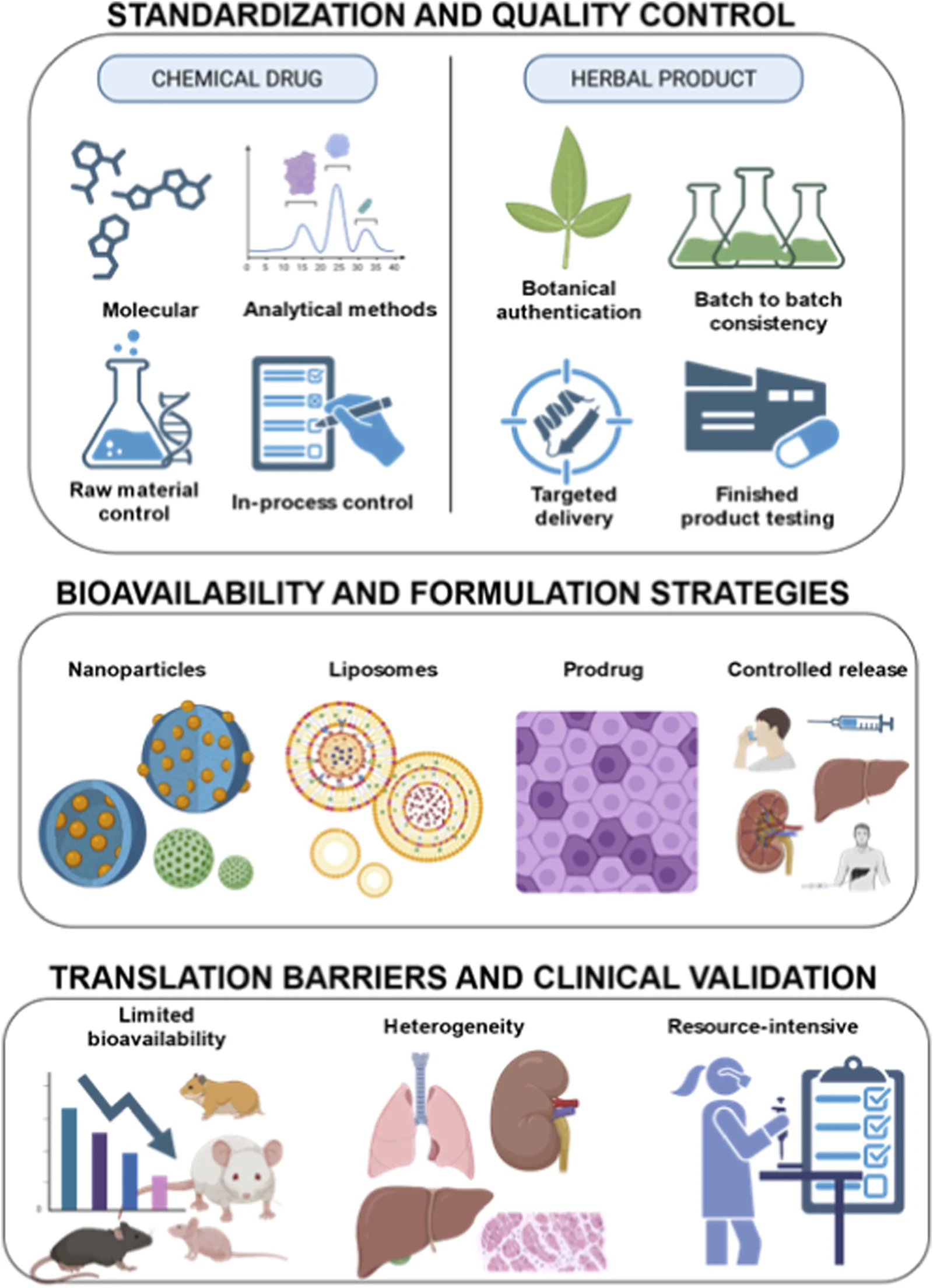
Schematic overview of standardization, quality control, bioavailability enhancement strategies, and translational barriers for chemical drugs versus herbal products. This infographic contrasts chemical drugs (left panels) with herbal products (right panels) in terms of standardization approaches encompassing molecular analytical methods and raw material control for chemicals, versus botanical authentication, batch-to-batch consistency, in-process controls, targeted delivery, and finished product testing for herbals (top section). The middle section illustrates bioavailability and formulation strategies, including nanoparticles, liposomes, and prodrugs for controlled release. The bottom section highlights key translational barriers such as limited bioavailability, heterogeneity, resource-intensive processes, raw material supply issues, preclinical/clinical validation challenges, and regulatory hurdles, emphasizing the complexities in developing herbal antifibrotic formulations for clinical translation.

The formulation of herbal drugs is important to ensure the quality and bioavailability of their active ingredients. Proper formulation of herbal drugs will improve their efficacy, dosing, and release, as well as patient compliance, thereby preserving the benefits of herbal medicines and developing them into safe and effective modern medicines (Figure 2).

### 6.1 Standardisation and quality control

Traditional medicine meets the healthcare needs of nearly 85% of the population worldwide, thereby emphasising the need to ensure the safety, quality, and efficacy of medicinal plants and their products to avoid serious health hazards (De Smet, 2002). Herbal product standardisation is divided into two types: (1) standardised extracts of known active constituents with proven therapeutic properties, and (2) marker-based extracts, in which the active constituent is unknown, and a specific marker compound is employed to standardise the quality (Hussain et al., 2009). Standardisation of herbal raw drugs requires a multidimensional approach that starts with compiling passport data, including medico-botanical surveys, accurate taxonomic identification, and botanical authentication. Macroscopic analysis (organoleptic evaluation) includes the evaluation of sensory properties such as shape, size, colour, texture, smell, and taste, whereas microscopic analysis (anatomical evaluation), aided by modern technologies such as light microscopy and scanning electron microscopy (SEM), helps in the accurate identification of herbal drugs and adulterants. Pharmacopoeial procedures facilitate detailed pharmacognostical evaluation, chromatographic identification, and physicochemical analysis to establish purity. Strength is determined by marker-based analysis or by estimating the active constituents that contribute to anti-fibrotic and anti-cancer properties. Safety assessments include heavy metal analysis, microbiological limit tests, aflatoxin and pesticide residue screening, and biological activity tests. Phytochemical standardisation also includes preliminary identification of chemical classes, estimation of bioactive classes (alkaloids, phenolics, triterpenoids, and tannins), and the creation of single and multi-marker fingerprint profiles to ensure consistency, efficacy, and quality (Nikam et al., 2012). In fibrosis, because small variations in the active ingredients may affect bioavailability or mechanism of action, high-quality standardisation is required (Kreutzer et al., 2022). Fibrosis drugs also have unique issues, such as the complexity of herbal anti-fibrotic drugs, which require precise marker selection (Bae et al., 2017). Additionally, biological drugs such as cell therapies and extracellular vesicles require lot-specific quality control measures, including sterility, identity, and potency testing. The sophisticated delivery vehicles, such as nanocarriers, also require quality-control tests that specifically target particle size, surface charge, and encapsulation efficiency (Wen et al., 2025). The development of high-quality, standardised quality criteria will be essential to ensuring the formulation’s authenticity and facilitating its preclinical evaluation and potential application in the treatment of fibrotic and cancer-related disorders.

### 6.2 Bioavailability and formulation strategies

Most anti-fibrotic drugs, natural compounds, peptides, and small molecules are challenged by low aqueous solubility and permeability, hindering their distribution to therapeutic levels in fibrotic tissues, which are shielded by thick ECM barriers (Lipinski, 2000). Poor bioavailability, coupled with issues such as first-pass hepatic metabolism, rapid enzymatic degradation (of peptides), and poor target specificity, can result in severely diminished therapeutic potency and increased risk of off-target toxicities (Gu et al., 2022). To address these issues, novel formulation technologies such as nanotechnology-based delivery systems, including solid lipid microparticles, polymeric nanoparticles, nanogels, and nanocrystals, are being investigated to improve the therapeutic potential of flavonoids in pulmonary fibrosis by protecting the drug from degradation, improving absorption, and facilitating tissue penetration (Sharma and Wairkar, 2024). Moreover, solubilization technologies such as amorphous solid dispersions, lipid-based formulations, and supersaturated solutions have been demonstrated to be effective in improving bioavailability (Di Costanzo and Angelico, 2019).

Prodrug strategies are used to transform drugs into more bioavailable compounds that selectively activate at the fibrotic target site, while controlled-release formulations, such as hydrogels and implants, offer sustained, localised drug delivery with reduced administration frequency (Han et al., 2022). Targeted delivery systems, including ligand-functionalized nanoparticles and peptide-conjugated carriers, further enhance site-specific accumulation by selectively binding to activated fibroblasts or ECM proteins. Alternative routes of administration, such as inhalation routes for pulmonary fibrosis (Xie et al., 2023), intraperitoneal or intrathecal routes, and topical routes for cutaneous fibrosis, can also circumvent systemic barriers and improve local efficacy. Future prospects include smart, stimuli-responsive systems that selectively release drugs based on local pH, enzyme activity, or redox state, along with combination therapies that combine anti-fibrotic drugs with immunomodulators or gene editing tools such as CRISPR. Personalized delivery systems designed to address individual fibrosis patterns are an exciting new frontier in overcoming bioavailability challenges and improving therapeutic outcomes in fibrotic disorders (Krajcer et al., 2025).

### 6.3 Translational barriers and clinical validation

Fibrosis is driven by a network of dysregulated cell populations, primarily myofibroblasts, along with contributions from epithelial, endothelial, and immune cells, which together sustain ECM accumulation and tissue remodelling. These cells exhibit persistent activation, resistance to apoptosis, and continuous secretion of pro-fibrotic mediators, collectively maintaining a self-perpetuating pathological microenvironment.

PUF offers a multi-target intervention by acting across these cellular compartments. Its phytoconstituents are reported to suppress myofibroblast activation, limit aberrant cellular transitions such as EMT, and modulate inflammatory cell signalling, thereby disrupting the cellular crosstalk that underlies fibrosis progression. This broad-spectrum activity distinguishes PUF from single-target therapies and supports its relevance in complex, multi-factorial diseases such as fibrosis and fibrosis-associated malignancies (Schuppan and Pinzani, 2012). However, despite this mechanistic rationale, clinical evidence supporting PUF remains insufficient. There are currently no well-defined clinical trials specifically evaluating PUF in fibrotic disorders, and available clinical data on related Unani formulations remain indirect and limited in scale (White et al., 2022; Zhao M. et al., 2022). This lack of direct validation restricts its acceptance within evidence-based therapeutic frameworks.

Progress toward clinical application is further constrained by standardization challenges, variability in bioactive composition, and limited pharmacokinetic characterization (Fuster-Martínez and Calatayud, 2024).

In addition, the absence of validated biomarkers complicates objective assessment of therapeutic outcomes in fibrosis (Zhao M. et al., 2022). Addressing these gaps will require focused translational strategies, including standardized formulation protocols, targeted preclinical validation in disease-relevant models, and rigorously designed clinical trials. Establishing safety, efficacy, and reproducibility will be essential to position PUF as a credible therapeutic candidate in fibrosis management (White et al., 2022).

## 7 Conclusion

### 7.1 Research gaps and overall recommendations

In summary, although herbal drugs have great potential as multi-targeted and safer alternatives for the management of fibrosis, their successful translation into evidence-based clinical practice remains constrained by several important scientific and translational challenges. These include the lack of standardization of raw materials, Polyherbal Unani Formulation (PUF) preparations, insufficient characterization of active phytochemical constituents, and limited understanding of the molecular mechanisms through which the complete formulation exerts its therapeutic effects. Standardization remains particularly challenging because therapeutic efficacy is likely to arise from the synergistic interactions among multiple bioactive constituents rather than from isolated compounds alone. Consequently, comprehensive phytochemical characterization, batch-to-batch quality control, and the identification of reliable marker compounds are essential to ensure formulation consistency and reproducibility. Furthermore, current evidence is derived predominantly from *in vitro* experiments and animal studies investigating individual herbal constituents, whereas mechanistic validation of the complete PUF remains limited. The lack of robust pharmacokinetic and pharmacodynamic data, together with insufficient clinical evidence regarding safety, efficacy, dosage optimization, and long-term therapeutic outcomes, continues to impede clinical translation. In addition, the absence of validated biomarkers and sensitive imaging modalities for monitoring disease progression and therapeutic response remains a major obstacle to the development and clinical evaluation of novel anti-fibrotic therapies (Patel and Sebastiani, 2020).

### 7.2 Summary of potential

Based on the available preclinical evidence, Polyherbal Unani Formulation (PUF) represents a promising multi-target therapeutic strategy for fibrotic disorders because of its chemically diverse bioactive constituents, which collectively modulate multiple molecular and cellular pathways involved in fibrosis. As discussed throughout this review, studies involving individual medicinal ingredients and their phytochemical constituents have consistently demonstrated antioxidant, anti-inflammatory, immunomodulatory, and anti-fibrotic activities through the regulation of key profibrotic pathways, including TGF-β/Smad, NF-κB, oxidative stress, extracellular matrix remodeling, and myofibroblast activation. These complementary mechanisms support the hypothesis that the complete PUF may exert synergistic therapeutic effects by simultaneously targeting several interconnected processes that drive fibrosis progression.

Importantly, while the mechanistic evidence supporting individual PUF constituents is substantial, direct experimental validation of the complete formulation remains comparatively limited. Therefore, the therapeutic potential of PUF should presently be interpreted within the context of predominantly preclinical evidence rather than established clinical efficacy. Nevertheless, its multi-target mode of action distinguishes PUF from conventional single-target therapeutic approaches and suggests potential applicability across a broad spectrum of fibrosis-associated disorders, including hepatic, pulmonary, renal, cardiac, and cancer-associated fibrosis. Future studies integrating standardized formulations, mechanistic validation, pharmacokinetic characterization, biomarker-guided therapeutic monitoring, and multi-omics approaches will be critical for defining the precise mechanisms of action of PUF and identifying patient populations most likely to benefit from treatment. Collectively, these advances will provide the scientific foundation necessary for well-designed clinical trials and facilitate the evidence-base clinical translation of PUF as a safe, effective, and scientifically validated anti-fibrotic therapeutic strategy.
